# Genetic Diversity and Host Specificity Varies across Three Genera of Blood Parasites in Ducks of the Pacific Americas Flyway

**DOI:** 10.1371/journal.pone.0116661

**Published:** 2015-02-24

**Authors:** Andrew B. Reeves, Mathew M. Smith, Brandt W. Meixell, Joseph P Fleskes, Andrew M. Ramey

**Affiliations:** 1 U.S. Geological Survey, Alaska Science Center, Anchorage, Alaska, United States of America; 2 U.S. Geological Survey, Western Ecological Research Center, Dixon Field Station, Dixon, California, United States of America; Linneaus University, SWEDEN

## Abstract

Birds of the order Anseriformes, commonly referred to as waterfowl, are frequently infected by Haemosporidia of the genera *Haemoproteus*, *Plasmodium*, and *Leucocytozoon* via dipteran vectors. We analyzed nucleotide sequences of the Cytochrome *b* (Cyt*b*) gene from parasites of these genera detected in six species of ducks from Alaska and California, USA to characterize the genetic diversity of Haemosporidia infecting waterfowl at two ends of the Pacific Americas Flyway. In addition, parasite Cyt*b* sequences were compared to those available on a public database to investigate specificity of genetic lineages to hosts of the order Anseriformes. Haplotype and nucleotide diversity of *Haemoproteus* Cyt*b* sequences was lower than was detected for *Plasmodium* and *Leucocytozoon* parasites. Although waterfowl are presumed to be infected by only a single species of *Leucocytozoon*, *L*. *simondi*, diversity indices were highest for haplotypes from this genus and sequences formed five distinct clades separated by genetic distances of 4.9%–7.6%, suggesting potential cryptic speciation. All *Haemoproteus* and *Leucocytozoon* haplotypes derived from waterfowl samples formed monophyletic clades in phylogenetic analyses and were unique to the order Anseriformes with few exceptions. In contrast, waterfowl-origin *Plasmodium* haplotypes were identical or closely related to lineages found in other avian orders. Our results suggest a more generalist strategy for *Plasmodium* parasites infecting North American waterfowl as compared to those of the genera *Haemoproteus* and *Leucocytozoon*.

## Introduction

Haemosporidia is a diverse order of intracellular parasites that infect vertebrates as their intermediate hosts. Although human malarial parasites, such as *Plasmodium falciparum* and *P*. *vivax*, are perhaps the most thoroughly researched and widely recognized taxa, haemosporidians have also been extensively studied in birds, thus facilitating our collective understanding of the complex life cycles of these parasites in vertebrate hosts[1]. Of all the haemosporidian parasites known to infect wild birds, perhaps the most studied are those belonging to the genera *Haemoproteus*, *Plasmodium* and *Leucocytozoon*. Parasites of these genera are transmitted to birds by dipteran vectors, specifically biting midges, mosquitos and black flies, respectively [1]. Infection may lead to clinical disease and even mortality in avian hosts [2,3]. Most infections are believed to be relatively benign [4,5], but sub-lethal effects of haemosporidian parasites in wild avian hosts are not well-understood.

Since the discovery of avian haemosporidians in 1884, the characterization of blood parasites has been largely accomplished through microscopy. Using such methods, at least 206 morphospecies have been described in avian hosts of 23 orders [1]. The highest parasite diversity (86 morphospecies) has been described in songbirds (order Passeriformes); in contrast, parasite diversity appears to be much lower in other avian taxa such as waterfowl (order Anseriformes), for whom 13 morphospecies of haemosporidians have been identified [1]. Of these 13 morphospecies, nine belong to the genus *Plasmodium*, three to *Haemoproteus*, and one to *Leucocytozoon* [1,6]. All but four of these morphospecies (three belonging to the genus *Plasmodium* and one classified as *Haemoproteus*) have been previously described in North America [1,6].

Molecular methodologies are increasingly being used to characterize haemosporidian infections in wild birds [7], although application of molecular methods to investigate parasites in some host taxa, such as waterfowl, is still limited. Genetic data from the relatively few molecular studies of haemosporidians in North American waterfowl suggest that genetic diversity of Leucocytozoon parasites at the mitochondrial DNA (mtDNA) Cyt*b* gene may be greater than for *Haemoproteus* and *Plasmodium* parasites [8–10]. This result seemingly contradicts collective findings of microscopical investigations that have identified greater morphospecies diversity for the genera *Haemoproteus* and *Plasmodium* in Anseriformes birds.

Recent molecular studies of haemosporidian parasites in waterfowl also suggest that differences may exist in host ranges for parasites of different genera; such differences may be important with regard to parasite evolution and mechanisms of dispersal. *Plasmodium* Cyt*b* lineages identified in ducks, swans and geese were found to be identical to parasites detected in diverse taxa including passerines, cranes, raptors and shorebirds [8,9,11–13], whereas *Haemoproteus* parasite haplotypes identified in waterfowl have only rarely been detected in non-Anseriformes taxa [12]. *Leucocytozoon* lineages identified for waterfowl parasites appear to be constrained solely to this taxon [8,9,13]. As such, additional genetic data obtained from haemosporidian parasites infecting a broad diversity of waterfowl over a large geographic range may be useful for better understanding the current distribution and genetic diversity of *Haemoproteus*, *Plasmodium* and *Leucocytozoon* parasites and determining the potential for waterfowl to redistribute parasites along migratory routes and among avian species.

In this study, we: (1) assess genetic diversity among three genera of blood parasites using waterfowl samples collected from six species of ducks at locations on opposite ends of the Pacific Flyway in North America, specifically in Alaska and California, USA, and (2) investigate host specificity by incorporating our data into a meta-analysis using parasite lineages derived from numerous avian taxa in order to better understand the distribution of parasites infecting wild waterfowl across space, time and host species. Results of this study will provide valuable baseline data for assessing potential changes of haemosporidian diversity and host-specificity through time as well as informing future efforts aiming to integrate microscopical and molecular parasitology.

## Materials and Methods

### Sample acquisition

From October 2006 to January 2007, wings from 860 hunter-shot ducks were collected from locations within the San Joaquin and Sacramento valleys of California (various sampling locations near 37° 3′ N, 120° 51′ W, and 39° 31′ N, 122° 12′ W). Whole blood samples from 1,347 live-trapped ducks were collected from May to August, 2010 on the Minto Flats State Game Refuge in interior Alaska (various sampling locations near 64° 53′ N, 148° 46′ W). Live captures were conducted under the authority of U.S. Fish and Wildlife permit # MB789758 in accordance with methods approved by the U.S. Geological Survey Alaska Science Center Animal Care and Use Committee (#2011–5). Four species of ducks were sampled at both locations: American Green-winged Teal (*Anas crecca*), American Wigeon (*Anas americana*), Mallard (*Anas platyrhynchos*), and Northern Pintail (*Anas acuta*). In addition, Lesser Scaup (*Athya affinis*) blood was collected in Alaska and wings from Northern Shoveler (*Anas clypeata*) were collected in California. Samples were taken from males and females, and from hatch-year and after-hatch-year ducks at all locations.

### Hematozoa detection and identification

Host and parasite DNA were extracted simultaneously using DNeasy Blood and Tissue Kit (Qiagen, Valencia, CA) following the manufacturer’s protocol and eluted with 100μl elution buffer. DNA was extracted from muscle tissues of frozen wings from California ducks and from whole blood stored at-80°C from samples originating in Alaska. Amplification and visualization of a fragment of host Cytochrome oxidase I gene, following protocols described by Kerr et al. [14], was completed to verify the competency of the DNA extraction prior to molecular screening for hematozoa DNA. A nested PCR protocol described by Hellgren et al. [7] was used to screen samples for the presence of hematozoa mtDNA Cytochrome *b* (Cyt*b*) gene. This method uses multiple primer sets to distinguish between *Leucocytozoon* DNA and that of *Haemoproteus*/*Plasmodium*. All samples were tested twice, and visualized on agarose gels using GelRed (Biotium, Hayward, CA) nucleic acid stain.

Positive PCR products were purified using Exo-SapIT (USB Inc., Cleveland, OH) and sequenced using Sanger sequencing techniques. Cycle sequencing was performed with identical primers used for the final PCR along with BigDye Terminator version 3.1 mix (Applied Biosystems, Foster City, CA) and analyzed on an Applied Biosystems 3730xl automated DNA sequencer (Applied Biosystems, Foster City, CA). Unedited sequences were analyzed using Basic Local Alignment Search Tool (BLAST)[15] to confirm nested PCR results and distinguish infections of *Haemoproteus* from *Plasmodium*. Sequences were assembled, edited, and cropped to a maximum length as determined by bi-directional coverage using Sequencher version 4.7 (Gene Codes Corp., Ann Arbor, MI). Sequences containing ambiguous bases were removed from subsequent analyses. Resultant sequences were assembled, aligned and cropped to a common length of 386 base pairs (bp) for further analyses. Parasite lineages for which only short bi-directional reads were obtained (i.e. < 386bp of homologous DNA) were omitted from genetic analyses.

### Assessing molecular diversity in parasites

Haplotype frequency and distances for all hematozoa sequences were summarized in a median-joining minimum spanning network created using Network version 4.6 (available at www.fluxus-engineering.com). To further explore parasite diversity in sample collections, haplotype diversity (*H*), and nucleotide diversity (π) values were calculated for sequences originating from Alaska and California were calculated for each parasite genus using Arlequin 3.5.1.2 [16].

### Phylogenetic analysis

A single representative of each blood parasite haplotype found in our study (n = 48; GenBank accession numbers: KM386316-KM386363) was included along with 1,076 unique Cyt*b* lineages of, *Haemoproteus*, *Plasmodium* and *Leucocytozoon* available on the MalAvi public Database as of March-11–2014 [17] for a total analysis of 1,124 parasite haplotypes. Database sequences were selected if they were void of any nucleotide ambiguities within the 386bp region corresponding to our sequences and had complete coverage over this region of the Cyt*b* gene. All avian host orders and the continents of origin were downloaded, abbreviated, and appended to each corresponding lineage name for sequences meeting the above criteria. A Maximum Likelihood tree with 1,000 bootstrap replicates was calculated in MEGA version 6.06 [18] using a Tamura-Nei model to assess the relatedness of blood parasites from waterfowl relative to those identified in other taxa for each of the three genera (*Haemoproteus*, *Plasmodium* and *Leucocytozoon*).

## Results

### Hematozoa detection, identification of haplotypes, and molecular diversity

In total, 1,654 positive blood parasite infections were identified from 2,207 samples tested (Table 1). After removing sequences with ambiguities and those shorter than 386bp, 1,029 sequences were used for subsequent analyses. Of these, 684 blood parasite sequences originated from ducks sampled in Alaska and 345 sequences were derived from ducks in California (Table 1). Totals of 316, 200, and 513 sequences were identified as *Haemoproteus*, *Plasmodium*, and *Leucocytozoon* respectively (Table 1). The large number of samples apparently coinfected with more than one genetic lineage of *Leucocytozoon* was the primary reason for the reduction in number of parasite sequences used in analyses of genetic diversity and host range (data not shown).

**Table 1:** Summary of field samples collected by location and species, the number of confirmed positive infections (+), the percentage of samples identified as positive (in parentheses), and the corresponding number of sequences used (*n*) in diversity and phylogenetic analyses (i.e. sequences used = the number of sequences with bi-directional coverage of 386 base pairs and no ambiguous bases) are indicated.

Species	Field samples collected	*Haemoproteus*		*Plasmodium*		*Leucocytozoon*	
		+	*n*	+	*n*	+	*N*
**Alaska**	**1,347**	**282 (21%)**	**240**	**179 (13%)**	**168**	**588 (44%)**	**276**
Lesser Scaup	60	16 (27%)	13	3 (5%)	3	33 (55%)	23
Mallard	481	112 (23%)	94	40 (8%)	38	173 (36%)	85
Northern Pintail	490	83 (17%)	73	114 (23%)	108	224 (46%)	117
American Green-winged Teal	228	30 (13%)	25	16 (7%)	14	84 (37%)	35
American Wigeon	88	41 (47%)	35	6 (7%)	5	74 (84%)	16
**California**	**860**	**88 (10%)**	**76**	**34 (4%)**	**32**	**483 (56%)**	**237**
Mallard	200	11 (6%)	10	8 (4%)	7	26 (13%)	12
Northern Pintail	60	2 (3%)	2	1 (2%)	1	35 (58%)	18
Northern Shoveler	200	14 (7%)	13	11 (6%)	10	118 (59%)	59
American Green-winged Teal	200	39 (20%)	34	5 (3%)	5	152 (76%)	75
American Wigeon	200	22 (11%)	17	9 (5%)	9	152 (76%)	73
**Total**	**2,207**	**370 (17%)**	**316**	**213 (10%)**	**200**	**1,071 (49%)**	**513**

A total of 48 unique hematozoa haplotypes, “DUCK01–DUCK48,” were identified in our samples: eight *Haemoproteus*, seven *Plasmodium*, and 33 *Leucocytozoon*. The two most common *Haemoproteus* haplotypes (DUCK01 = 81% and DUCK02 = 17% of sequences) were detected in samples of all six duck species sampled, whereas the six remaining haplotypes were identified in only a single sample each (Table 2). Similarly, the two most common *Plasmodium* haplotypes (DUCK09 = 72% and DUCK10 = 26% of sequences) were detected in six and five species of ducks, respectively, in contrast to the other five haplotypes, each being detected in a single sample (Table 2). Eighty-nine percent of the *Leucocytozoon* sequences were distributed across the seven most common haplotypes with the two most common haplotypes representing the majority of *Leucocytozoon* infections (41% in DUCK19 and 15% in DUCK18). Fourteen of 33 *Leucocytozoon* haplotypes were detected in more than one species of duck (Table 2).

**Table 2:** Summary of haplotypes identified in this study, the corresponding frequency at which they were identified, and host species within which they originated (L = Lesser Scaup, M = Mallard, N = Northern Pintail, S = Northern Shoveler, T = American Green-winged Teal, W = American Wigeon).

Detection in Pacific Americas Flyway Ducks					BLAST results			
	Haplotype	Accession	Frequency total	Species of origin	Accession	Identity	MalAvi lineage	Avian host/vector
*Haemoproteus*	DUCK01	KM386316	255	LMPSTW	JQ314226	100	TUSW07	*Cygnus columbianas*
	DUCK02	KM386317	55	LMPSTW	JQ314225	100	TUSW06	*Cygnus columbianas*
	DUCK03	KM386318	1	P	JQ314226	99	TUSW07	*Cygnus columbianas*
	DUCK04	KM386319	1	M	JQ314226	99	TUSW07	*Cygnus columbianas*
	DUCK05	KM386320	1	M	JQ314226	99	TUSW07	*Cygnus columbianas*
	DUCK06	KM386321	1	L	JQ314225	99	TUSW06	*Cygnus columbianas*
	DUCK07	KM386322	1	P	JQ314225	99	TUSW06	*Cygnus columbianas*
	DUCK08	KM386323	1	M	JQ314226	99	TUSW07	*Cygnus columbianas*
*Plasmodium*	DUCK09	KM386357	144	LMPSTW	HF543643	100	BT7	*Milvus sp*
	DUCK10	KM386358	51	MPSTW	AB741489	100	*Mallard partial*	*Anas platyrhynchos*
	DUCK11	KM386359	1	P	DQ368392	100	SYBOR2	*Sylvia borin*
	DUCK12	KM386360	1	L	KC867673	100	BOBOVT	*Dolichonyx oryzivorus*
	DUCK13	KM386361	1	L	KC867677	100	BOBO	*Dolichonyx oryzivorus*
	DUCK14	KM386362	1	T	AB741489	99	*Anas platyrhynchos 4*	*Anas platyrhynchos*
	DUCK15	KM386363	1	W	HF543643	99	ENV	*Milvus sp*
*Leucocytozoon*	DUCK16	KM386324	38	MPSTW	JQ314224	100	TUSW05	*Cygnus columbianas*
	DUCK17	KM386325	3	MS	JQ314224	99	TUSW05	*Cygnus columbianas*
	DUCK18	KM386326	78	MPTW	KC409122	100	NOPI04	*Anas acuta*
	DUCK19	KM386327	208	LMPSTW	JQ314223	100	TUSW04	*Cygnus columbianas*
	DUCK20	KM386328	3	M	KC409123	99	NOPI05	*Anas acuta*
	DUCK21	KM386329	69	MPTW	AB743872	100	*Anas crecca*	*Anas crecca*
	DUCK22	KM386330	8	MPTW	AB743872	99	*Anas crecca*	*Anas crecca*
	DUCK23	KM386331	17	MPT	AB741516	99	*Cygnus cygnus*	*Cygnus cygnus*
	DUCK24	KM386332	36	MP	KC409123	100	NOPI05	*Anas acuta*
	DUCK25	KM386333	12	LS	HF543635	96	MILVUS02	*Milvus sp*
	DUCK26	KM386334	2	P	KC409122	99	NOPI04	*Anas acuta*
	DUCK27	KM386335	1	P	KC409122	99	NOPI04	*Anas acuta*
	DUCK28	KM386336	1	P	JQ314223	99	TUSW04	*Cygnus columbianas*
	DUCK29	KM386337	1	M	EF032867	99	IHELLI1	Black fly, vector
	DUCK30	KM386338	1	L	EF032867	100	IHELLI1	Black fly, vector
	DUCK31	KM386339	1	L	JQ314223	99	TUSW04	*Cygnus columbianas*
	DUCK32	KM386340	1	P	AB743872	99	*Anas crecca*	*Anas crecca*
	DUCK33	KM386341	1	L	HF543635	96	MILVUS02	*Milvus sp*
	DUCK34	KM386342	1	T	AB743872	99	*Anas crecca*	*Anas crecca*
	DUCK35	KM386343	1	M	KC409123	99	NOPI05	*Anas acuta*
	DUCK36	KM386344	2	PW	JQ314223	99	TUSW04	*Cygnus columbianas*
	DUCK37	KM386345	6	MPTW	KC409119	100	NOPI01	*Anas acuta*
	DUCK38	KM386346	1	P	AB743872	99	*Anas crecca*	*Anas crecca*
	DUCK39	KM386347	2	T	AB743872	99	*Anas crecca*	*Anas crecca*
	DUCK40	KM386348	7	TW	KC409127	100	NOPI09	*Anas acuta*
	DUCK41	KM386349	4	TW	KC409126	100	NOPI08	*Anas acuta*
	DUCK42	KM386350	2	W	KC409119	99	NOPI01	*Anas acuta*
	DUCK43	KM386351	1	S	KC409119	99	NOPI01	*Anas acuta*
	DUCK44	KM386352	1	P	JQ314223	99	TUSW04	*Cygnus columbianas*
	DUCK45	KM386353	1	T	JQ314223	99	TUSW04	*Cygnus columbianas*
	DUCK46	KM386354	1	T	JQ314223	99	TUSW04	*Cygnus columbianas*
	DUCK47	KM386355	1	W	KC409119	99	NOPI01	*Anas acuta*
	DUCK48	KM386356	1	W	AB741516	99	*Cygnus cygnus 4*	*Cygnus cygnus*

The highest scoring NCBI BLAST results are given right of vertical dotted line for each haplotype along with the corresponding accession number, identity and the MalAvi lineage. The sequence name in italics corresponding to the accession number is provided if no MalAvi lineage was identified in conjunction with the highest scoring BLAST result. The origin (avian host or arthropod vector) of the most closely related parasite cytochrome *b* sequence as inferred by the highest scoring maximum identity score is provided in the far-right column.

**Fig 1:**
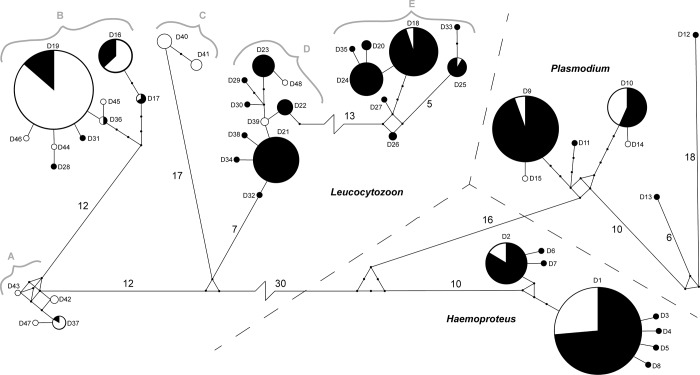
Minimum spanning network constructed from 48 haplotypes of a 386 base pair region of the Cytochrome *b* gene for haemosporidian parasites detected in ducks of the Pacific Americas Flyway. Circles are drawn proportional to the frequency at which the haplotypes were observed. Shading of circles represents the proportional geographic origin of samples (black = Alaska; white = California). Line segment lengths are proportional to the number of nucleotide differences between haplotypes unless indicated by a line break. Small dots are used to indicate predicted but undetected haplotypes closely related to our data (<5 mutations) while numbers adjacent to line segments correspond to larger distances (≥5 mutations). The three genera of blood parasites are separated by dashed lines. Haplotype names as they appear in Table 2 have been abbreviated here (e.g. D4 = DUCK04). Gray letters and brackets correspond to *Leucocytozoon* clades identified in Fig. 5.

All eight *Haemoproteus* haplotypes were identified in blood samples collected from ducks in Alaska; in contrast only the two most common haplotypes were detected in tissues originating from waterfowl sampled in California (Fig. 1). Five of eight *Plasmodium* haplotypes were detected in Alaska samples as compared to four haplotypes detected in California. All *Plasmodium* haplotypes detected in more than one sample were found in both locations (Fig. 1). Of the 33 *Leucocytozoon* haplotypes identified in our samples, 23 were detected in Alaska and 17 in California (Fig. 1). Seven *Leucocytozoon* haplotypes were detected at both locations in contrast to 26 haplotypes that were unique to parasites in ducks sampled at only one location; these included some of the more common haplotypes (e.g. DUCK21 = 13% and DUCK24 = 7% of sequences) that were only detected in Alaska.

The calculated *H* and π for parasites from the six duck species sampled was lowest in *Haemoproteus* and highest in *Leucocytozoon* (Table 3). Differences in *H*, but not for π, were observed for parasites identified in sample collections from Alaska and California for all genera of blood parasites (Table 3).

**Table 3:** Haplotype and nucleotide diversity values (H and π respectively) and their 95% confidence intervals for all haplotypes belonging to each genus of haemosporidian identified in this study.

	*H*	π
*Haemoproteus*	0.320 +/- 0.029	0.003 +/- 0.002
*Plasmodium*	0.419 +/- 0.032	0.010 +/- 0.006
*Leucocytozoon*	0.783 +/-0.014	0.045 +/- 0.022

All calculations performed in Arlequin 3.5.1.2 [16].

### Phylogenetic relatedness of parasites from different avian host species

**Fig 2:**
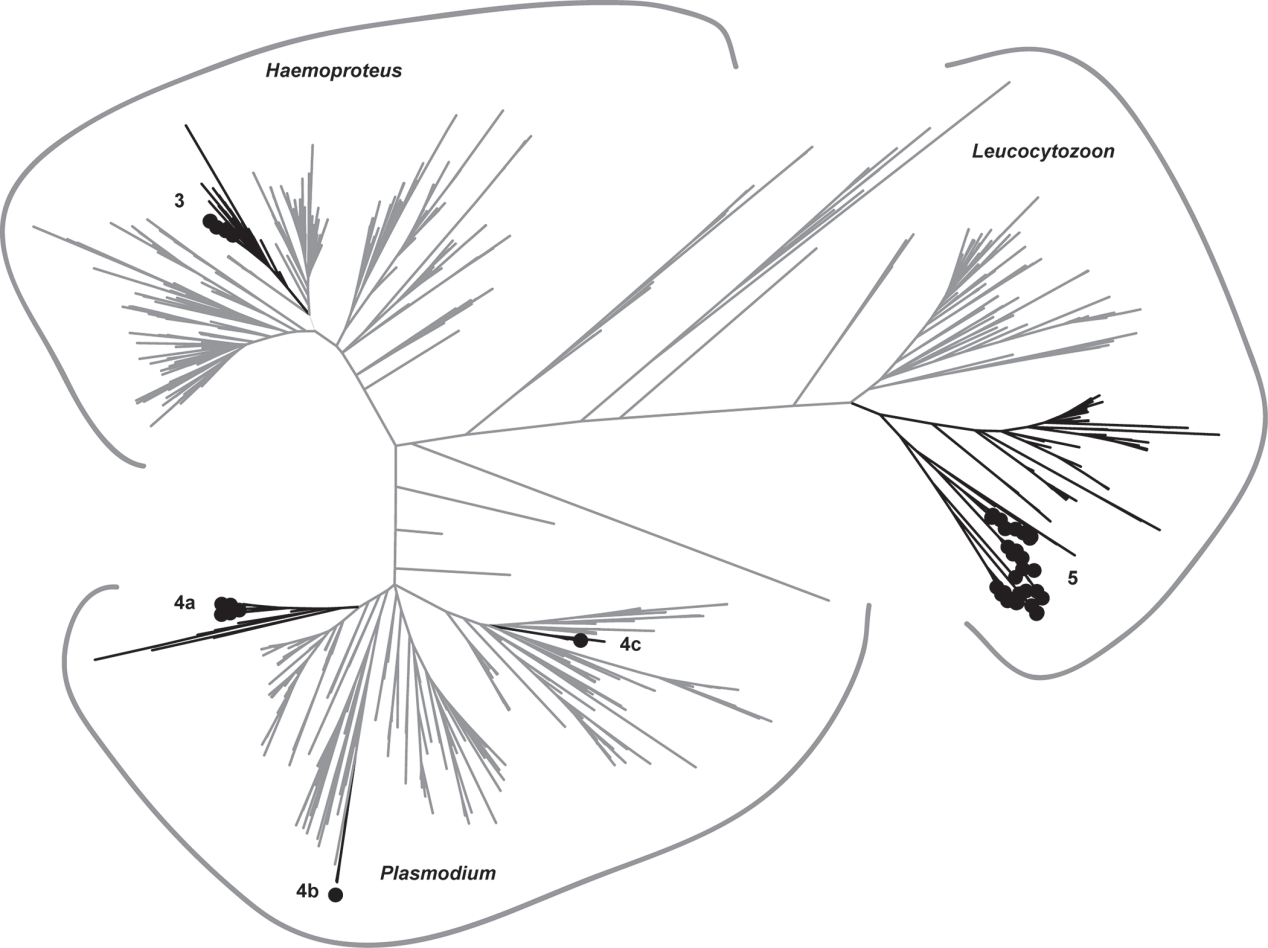
Maximum likelihood phylogenetic analysis of 1,124 haemosporidian sequences constructed from 386 base pairs of the Cytochrome *b* gene. Sequences detected in Pacific Americas Flyway ducks in this study are labeled with black circles; lineages (branches) that appear in sub-trees (Figs. 3–5) are shaded black.

The 1,124 sequences in the ML tree clustered by genera as expected (Fig. 2, S1 Fig.). Large clades generally had poor bootstrap support. However, terminal clades containing sequences originating from waterfowl had higher support (>50) with few exceptions.

#### Haemoproteus

**Fig 3:**
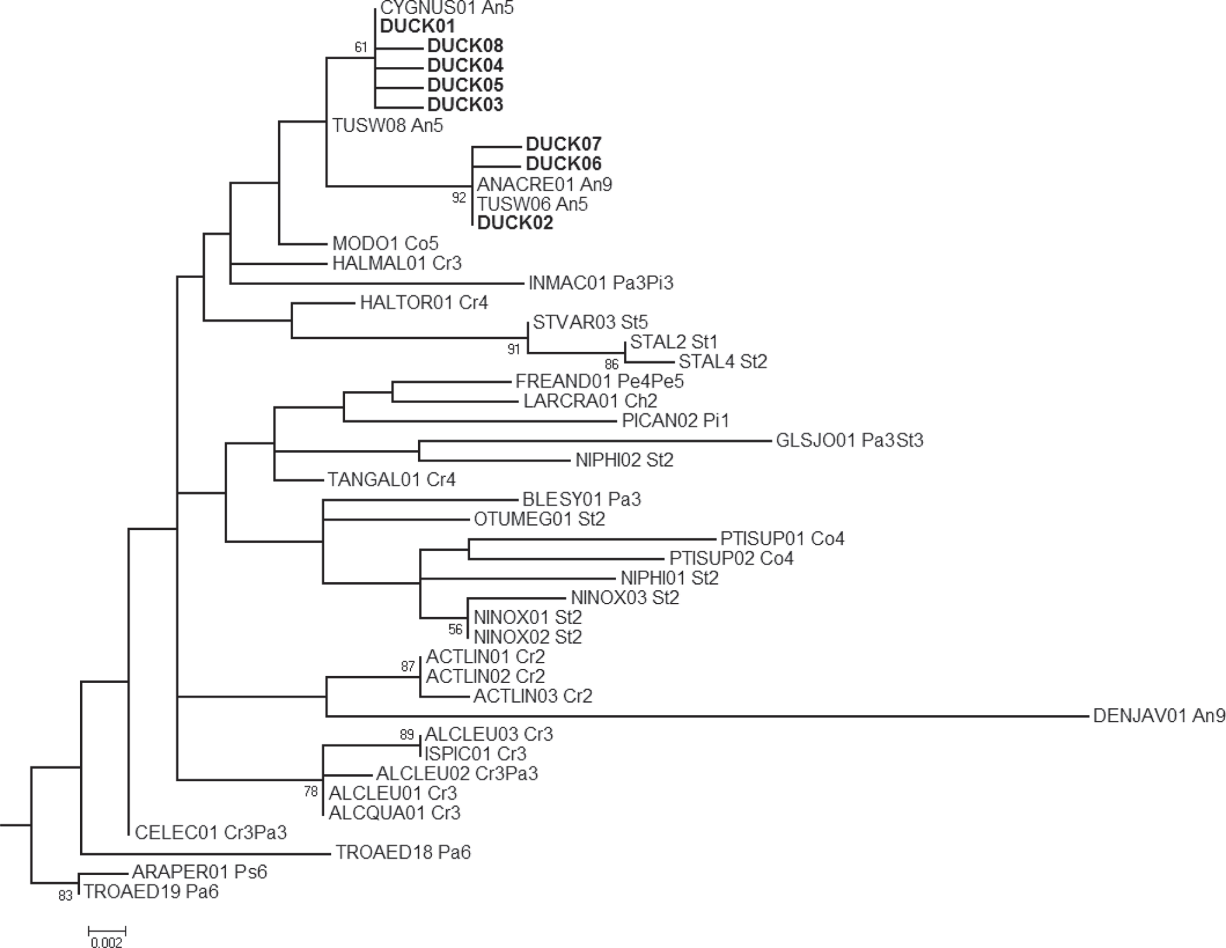
*Haemoproteus* sub-tree from a Maximum likelihood phylogenetic analysis of haemosporidian sequences constructed from 386 base pairs of the Cytochrome *b* gene. Bootstrap values (1,000 replicates) >50 are shown at nodes. Haplotypes derived from Alaska and California duck samples for this study (DUCK01–DUCK08) are shown in bold. Two-letter codes following the lineage names of MalAvi acquired sequences refer to the avian order/s reported as the host origin: Anseriformes (An), Bucerotiformes (Bu), Charadriiformes (Ch), Columbiformes (Co), Coraciiformes (Cr), Galliformes (Ga), Passeriformes (Pa), and Strigiformes (St). Numbers following the order code refer to the reported continent of origin: Europe (1), Asia (2), Africa (3) Oceania (4), North America (5), South America (6) and unknown (9).

All *Haemoproteus* haplotypes from sequences originating in ducks from Alaska and California were closely related and nested within a single clade in the ML tree (Figs. 2 and 3). Four lineages, of Anseriformes origin, identified from the MalAvi database were also embedded in this clade, and three of those four lineages were identical to sequences from Alaska and California. Two *Haemoproteus* haplotypes identified from waterfowl samples as reported on the MalAvi database were phylogenetically divergent from this clade: DENJAV01 (Fig. 3) and WW1 (S1 Fig.). DENJAV01 and WW1 were related to lineages infecting Coraciiformes and Passeriformes in Eurasia respectively.

#### Plasmodium

**Fig 4:**
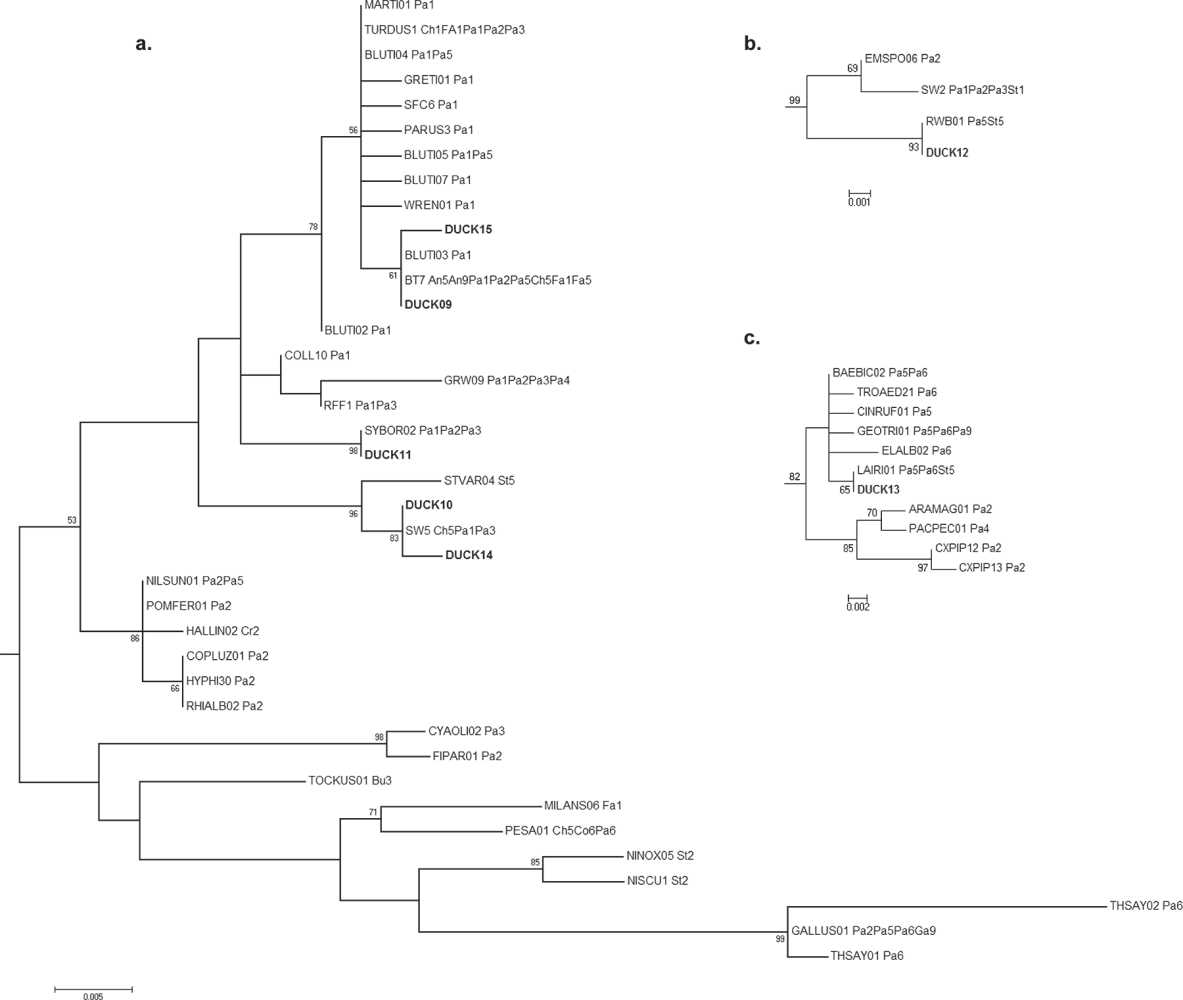
*Plasmodium* sub-trees from a Maximum likelihood phylogenetic analysis of haemosporidian sequences constructed from 386 base pairs of the Cytochrome *b* gene. Bootstrap values (1,000 replicates) >50 are shown at nodes. Haplotypes derived from Alaska and California duck samples for this study (DUCK09–DUCK15) are shown in bold. Two-letter codes following the lineage names of MalAvi acquired sequences refer to the avian order/s reported as the host origin: Anseriformes (An), Bucerotiformes (Bu), Charadriiformes (Ch), Columbiformes (Co), Coraciiformes (Cr), Falconiformes (Fa), Passeriformes (Pa), Pelicaniformes (Pe), Piciformes (Pi), Psittaciformes (Ps), and Strigiformes (St). Numbers following the order code refer to the reported continent of origin: Europe (1), Asia (2), Africa (3) Oceania (4), North America (5), South America (6) and unknown (9).

Five of the seven *Plasmodium* haplotypes identified in Alaska and California ducks were embedded in a clade comprised of haplotypes originating in Anseriformes and four additional orders of birds (Charadriiformes, Falconiformes, Passeriformes, and Strigiformes; Figs. 2 and 4A). The remaining two *Plasmodium* haplotypes (DUCK12–13) identified in our data originated from Lesser Scaup samples collected in Alaska, and were found in two distantly related clades comprised of three and ten MalAvi lineages respectively (Figs. 2 and 4B-C). The sequences in these clades, including identical lineages to DUCK12 and DUCK13, originated from parasites detected in Passeriformes and Strigiformes.

#### Leucocytozoon

**Fig 5:**
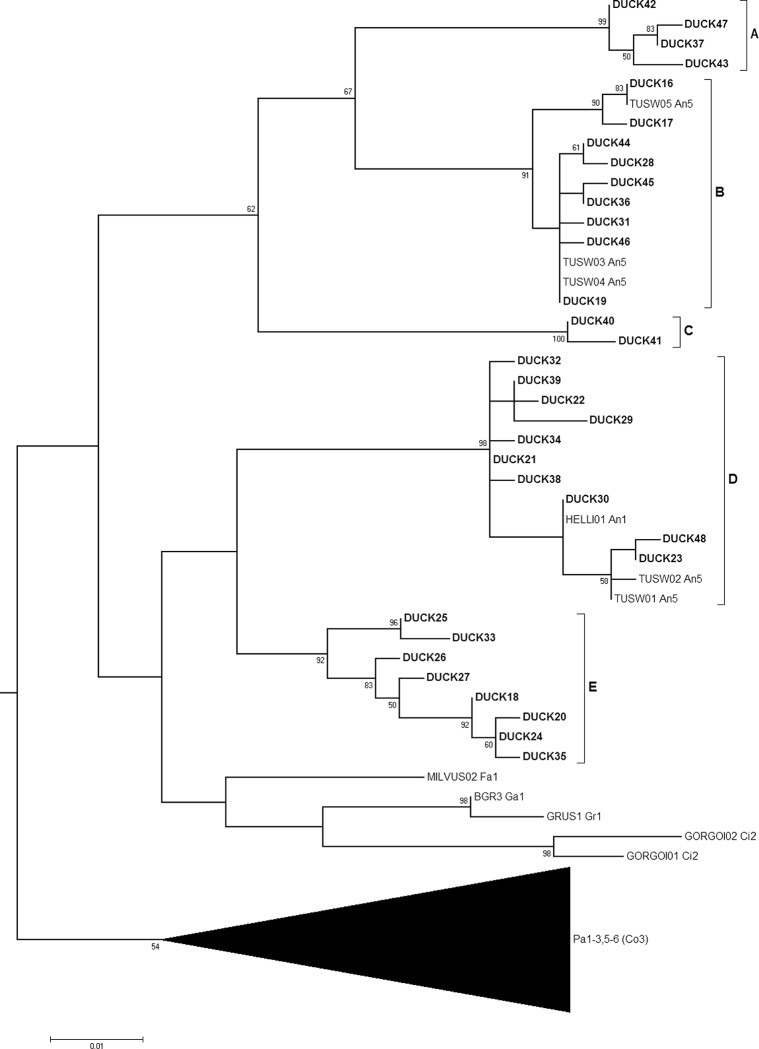
*Leucocytozoon* sub-tree from a Maximum likelihood phylogenetic analysis of haemosporidian sequences constructed from 386 base pairs of the Cytochrome *b* gene. Bootstrap values (1,000 replicates) >50 are shown at nodes. Haplotypes derived from Alaska and California duck samples for this study (DUCK16–DUCK48) are shown in bold. Two-letter codes following the lineage names of MalAvi acquired sequences refer to the avian order/s reported as the host origin: Anseriformes (An), Ciconiiformes (Ci), Columbiformes (Co), Falconiformes (Fa), Galliformes (Ga), Gruiformes (Gr), and Passeriformes (Pa). Numbers following the order code refer to the reported continent of origin: Europe (1), Asia (2), Africa (3) Oceania (4), North America (5), South America (6) and unknown (9). The black triangle at the bottom of the tree represents a collapsed view of a large clade comprised of 120 lineages, 119 of those originating in passerines from all geographic regions of the world, except Oceania. The Columbiformes code (Co) appears in parentheses as this avian order was only attributed to one lineage in this clade.

All 33 *Leucocytozoon* haplotypes originating from ducks in Alaska and California clustered in a single clade within the ML tree (Fig. 2). These *Leucocytozoon* haplotypes formed 5 well supported clades (“A”–“E” in Fig. 5; bootstrap > 91), each separated by distances of 4.9%–7.6% via post-hoc calculation of pairwise differences (Table 4) performed in Arlequin 3.5.1.2 [16]. Six lineages originating in Anseriformes were identified from the MalAvi database, all of which were embedded in either clade B or D (Fig. 5). Clades D and E can be described, via the topology of the ML tree, as having closer shared ancestry with a clade comprised of parasite lineages originating from non-waterfowl avian orders (i.e. MILVUS02, BGR3, GRUS1, GORGOI01, GORGOI02) than with clades A–C; however, support for this inferred relationship was weak (bootstrap = 5; S1 Fig.).

**Table 4:** Genetic distances between clades of *Leucocytozoon* haplotypes sequenced in this study.

	Leuc A	Leuc B	Leuc C	Leuc D	Leuc E
Leuc A	**0.004 +/- 0.003**				
Leuc B	0.050*	**0.004 +/- 0.003**			
Leuc C	0.065*	0.058*	**0.003 +/- 0.002**		
Leuc D	0.062*	0.070*	0.068*	**0.004 +/- 0.003**	
Leuc E	0.076*	0.074*	0.073*	0.049*	**0.006 +/- 0.004**

Average number of **pa**irwise differences between individuals within populations (PiX)/386bp are given as nucleotide diversity index on the diagonal in bold. Average numbers of pairwise differences between populations (PiXY)/386bp are given below diagonal. Asterisks indicate values with highly significant P-values (<0.001). All calculations performed in Arlequin 3.5.1.2 [16].

## Discussion

### Genetic diversity

It is generally accepted that *L*. *simondi* is the only species of *Leucocytozoon* infecting waterfowl [1]. Interestingly, the genetic diversity of *Leucocytozoon* parasites sampled from waterfowl in this study was higher than that found in either *Haemoproteus* or *Plasmodium*. Assuming all sequences produced in this study are from *L*. *simondi*, the genetic distances observed (up to 7.6%) among the five clades are relatively large for a single taxon. By comparison the average genetic distance between Cyt*b* sequences from all recognized avian *Haemoproteus* species in the MalAvi database is 7.0% [19] and the average genetic distance between haplotypes of different genera (*Haemoproteus* and *Plasmodium*) derived from Alaska and California ducks was 8.9% (data not shown). It is likely that multiple species of *Leucocytozoon* are represented here, or perhaps cryptic species are present but not yet identified through morphological examination of infected erythrocytes. A thorough study combining traditional morphometric taxonomy with genetic analyses would be useful to assess the possibility of cryptic speciation. This type of study has been conducted before to distinguish species previously thought to comprise a single morphospecies (i.e. genetic clades of *L*. *toddi* with 10.9% divergence infecting different taxa of diurnal raptors; [20]).

Compared to *Leucocytozoon*, a lower level of genetic diversity was observed among *Haemoproteus* sequences originating in Alaska and California waterfowl. Two species of *Haemoproteus* parasites (*H*. *nettions and H*. *greineri*) have previously been reported to infect North American waterfowl, however, the possibility that these species may be synonymous has also been proposed [1]. The low genetic diversity of *Haemoproteus* parasites observed in Alaska and California waterfowl supports a single species hypothesis, although a detailed study pairing morphological analysis of parasites with genetic data is necessary to properly investigate this postulate. We cannot ascertain as to whether both morphospecies are represented within the samples tested in the current study despite a large sample size from two geographically distant areas.

Similar to *Haemoproteus* parasites, a relatively low genetic diversity was also observed for *Plasmodium*, despite previous reports of up to six species of parasites of this genera infecting North American waterfowl [1]. Several factors may at least partially explain this result. Unlike *Leucocytozoon* parasites which are readily detected in avian muscle tissue [9], the nested PCR used in this study may not be as sensitive for *Plasmodium* parasites; which may account for the detection of relatively few parasites of this genus in California. Prevalence may also be lower for *Plasmodium* parasites in waterfowl in the Pacific Flyway as compared to the other haemosporidian genera investigated. The detection of multiple divergent *Plasmodium* haplotypes, however, is consistent with infection of waterfowl in Alaska and California with multiple *Plasmodium* species.

Haplotypes frequently detected in parasites from both Alaska blood and California wing samples (e.g. *Haemoproteus* DUCK01 and DUCK02; Fig. 1), may indicate these lineages are widespread across western North America and may be redistributed along the Pacific Americas Flyway in migratory birds. On the other hand, unique haplotypes identified in multiple samples at only one location (e.g. DUCK21 and DUCK40; Fig. 1), may indicate differences among sample collections. Disproportional sharing of some of the more frequently observed *Plasmodium* and *Leucocytozoon* haplotypes provide further evidence for differences among sample collections. For example, the majority of *Plasmodium* sequences from Alaska samples (81%) were identified as haplotype DUCK09; while 69% of sequences originating from California samples were DUCK10 (Fig. 1). *Leucocytozoon* sequences for parasites originating in California were predominate in clades A, B, and C while clades D and E were comprised almost exclusively of parasite lineages detected in Alaska (Fig. 1). Possible explanations for these observed differences are numerous. Although all six species of ducks in this study have some degree of migratory connectivity between Alaska and California, the Central Valley wintering grounds of California receive a larger total number of annual migrants (e.g. Northern Pintails, American Green-winged Teal and American Wigeon) from prairie pothole breeding grounds in north-central North America than from Alaska [21]. Therefore, assemblages of hosts in California from multiple breeding locations may contribute to differences in genetic diversity of parasites detected in the two sampling locations. Furthermore, Lesser Scaup and Northern Shoveler were not sampled at both locations, and sample collections from Alaska and California were obtained in different seasons, years, and from dissimilar tissue types. Thus confounding precludes robust inference on whether genetic differences observed between sample collections for *Plasmodium* and *Leucocytozoon* parasites are a function of space, time, detection biases, host species or a combination thereof.

### Host specificity

Although the detection of the two most common *Haemoproteus* haplotypes in all six species of ducks sampled as part of this study suggests parasite sharing among waterfowl species, the clustering of all eight *Haemoproteus* haplotypes detected in samples collected from Alaska and California within a monophyletic clade along with four waterfowl-origin lineages obtained from the MalAvi database suggests a relatively narrow host range (Anseriformes) for these closely related parasites. The reporting of two additional phylogenetically divergent *Haemoproteus* lineages as infecting waterfowl on the MalAvi database, DENJAV01 and WW1, closely related to lineages infecting Coraciiformes and Passerines, respectively, suggests that these parasite lineages may be the result of spillover from non-waterfowl hosts. Collectively, these findings are consistent with previous studies suggesting that *Haemoproteus* species are more likely to be specialized to hosts than *Plasmodium* [4,11,22]; however, they also provide evidence for infrequent host switching for *Haemoproteus* parasites.

*Plasmodium* haplotypes detected in more than one individual in Alaska or California were also detected in at least five species of ducks, suggesting parasite sharing among waterfowl. However, *Plasmodium* also showed considerably more evidence for host switching compared to other genera. The clustering of five *Plasmodium* haplotypes detected in Alaska and California ducks in a clade with 18 additional lineages identified in Passeriformes, Charadriiformes, Falconiformes, and Strigiformes provides evidence for repeated cross species transmission of parasites in the presence of suitable vectors. Similarly, the shared identity and close phylogenetic relationship of two divergent *Plasmodium* haplotypes identified in our study, with lineages originating from Passeriformes and Strigiformes, provides further evidence for relatively frequent host switching for parasites of this genus. A generalist strategy of parasitism for *Plasmodium* in wild birds is also supported by previous molecular studies [11,22].

In contrast to *Plasmodium*, the exclusive relationship between waterfowl and *Leucocytozoon* Cyt*b* lineages supports a relatively high degree of host specificity for parasites of this genus with respect to Anseriformes birds. Inferred shared ancestry of Clades D-E with parasite lineages detected in non-waterfowl orders may indicate previous host switching events; however the limited support for this relationship may also suggest that the phylogenetic analysis failed to identify the true ancestral relationship. Thus whether or not all *Leucocytozoon* lineages detected in waterfowl have common, monophyletic ancestry and evolved with Anseriformes hosts remains uncertain. Such a scenario would be supported by experimental studies that provide evidence for host specificity of *L*. *simondi* to waterfowl [23,24].

### Conclusions and future work

This study provides evidence that genetic diversity and host specificity vary for three genera of haemosporidian parasites in ducks of the Pacific Americas Flyway. Based on these results we identified three topics of future research direction. First, the relatively high genetic diversity of *Leucocytozoon* parasites, and low diversity of *Haemoproteus* parasites occurring in waterfowl highlights the need for future research that combines microscopy and molecular techniques to reassess taxonomic speciation within these genera. Such an assessment would improve inference of future investigations aiming to examine parasite evolution and pathogenic effects on hosts. Second, the frequency of coinfections in our samples, particularly the number of ducks with multiple variants of *Leucocytozoon*, suggests that intra-host parasite interactions may play a role in infection dynamics in waterfowl as has been shown for other avian taxa [25]. Future investigations examining parasite competition and effects of coinfections on host species may be helpful for understanding observed differences in parasite diversity and factors contributing to pathogenicity. Third, the observed differences in haplotype frequencies between sample collections from Alaska and California warrant further investigation of temporal, regional, and tissue-specific differences in parasite distributions. Controlling the number of variables between the two locations (i.e. species, time, and tissue type) will increase the resolution of results and facilitate a better understanding of how birds may redistribute parasites through migration.

## Supporting Information

S1 Fig Maximum likelihood phylogenetic analysis of 1,124 haemosporidian sequences constructed from 386 base pairs of the cytochrome *b* gene. Bootstrap values (1,000 replicates) are shown at nodes. Haplotypes derived from Alaska and California duck samples for this study (DUCK01–DUCK48) are shown in bold. A single-letter code preceding the lineage names of MalAvi acquired sequences refers to the parasite genus: *Haemoproteus* (H), *Plasmodium* (P) and *Leucocytozoon* (L). Two-letter codes following the lineage names of MalAvi acquired sequences refer to the avian order/s reported as the host origin: Anseriformes (An), Apodiformes (Ap), Apterygiformes (At), Bucerotiformes (Bu), Charadriiformes (Ch), Ciconiiformes (Ci), Columbiformes (Co), Caprimulgiformes (Cp), Coraciiformes (Cr), Cuculiformes (Cu), Falconiformes (Fa), Galliformes (Ga), Galbuliformes (Gl), Gruiformes (Gr), Gaviiformes (Gv), Passeriformes (Pa), Pelicaniformes (Pe), Piciformes (Pi), Phoenicopteriformes (Pn), Procellariiformes (Pr), Psittaciformes (Ps), Sphenisciformes (Sp), Stigiformes (St), Trochiliformes (To), and Upupiformes (Up). Numbers following the order code refer to the reported continent of origin: Europe (1), Asia (2), Africa (3) Oceania (4), North America (5), South America (6) and unknown (9). *P*. *falciparum* (AF069609) and *P*. *reichenowi* (AF069610) sequences were included as blood parasite references for non-avian hosts.
(PNG)
